# Aortic Wall Inflammation Predicts Abdominal Aortic Aneurysm Expansion, Rupture, and Need for Surgical Repair

**DOI:** 10.1161/CIRCULATIONAHA.117.028433

**Published:** 2017-08-28

**Authors:** The MARS Study Investigators

**Affiliations:** From Centre for Cardiovascular Science, Edinburgh, Scotland.

**Keywords:** abdominal aortic aneurysm, MRI, repair, rupture

## Abstract

**Background:**

Ultrasmall superparamagnetic particles of iron oxide (USPIO) detect cellular inflammation on magnetic resonance imaging (MRI). In patients with abdominal aortic aneurysm, we assessed whether USPIO-enhanced MRI can predict aneurysm growth rates and clinical outcomes.

**Methods:**

In a prospective multicenter open-label cohort study, 342 patients with abdominal aortic aneurysm (diameter ≥40 mm) were classified by the presence of USPIO enhancement and were monitored with serial ultrasound and clinical follow-up for ≥2 years. The primary end point was the composite of aneurysm rupture or repair.

**Results:**

Participants (85% male, 73.1±7.2 years) had a baseline aneurysm diameter of 49.6±7.7 mm, and USPIO enhancement was identified in 146 (42.7%) participants, absent in 191 (55.8%), and indeterminant in 5 (1.5%). During follow-up (1005±280 days), 17 (5.0%) abdominal aortic aneurysm ruptures, 126 (36.8%) abdominal aortic aneurysm repairs, and 48 (14.0%) deaths occurred. Compared with those without uptake, patients with USPIO enhancement have increased rates of aneurysm expansion (3.1±2.5 versus 2.5±2.4 mm/year, *P*=0.0424), although this was not independent of current smoking habit (*P*=0.1993). Patients with USPIO enhancement had higher rates of aneurysm rupture or repair (47.3% versus 35.6%; 95% confidence intervals, 1.1–22.2; *P*=0.0308). This finding was similar for each component of rupture (6.8% versus 3.7%, *P*=0.1857) or repair (41.8% versus 32.5%, *P*=0.0782). USPIO enhancement was associated with reduced event-free survival for aneurysm rupture or repair (*P*=0.0275), all-cause mortality (*P*=0.0635), and aneurysm-related mortality (*P*=0.0590). Baseline abdominal aortic aneurysm diameter (*P*<0.0001) and current smoking habit (*P*=0.0446) also predicted the primary outcome, and the addition of USPIO enhancement to the multivariate model did not improve event prediction (c-statistic, 0.7935–0.7936).

**Conclusions:**

USPIO-enhanced MRI is a novel approach to the identification of aortic wall cellular inflammation in patients with abdominal aortic aneurysms and predicts the rate of aneurysm growth and clinical outcome. However, it does not provide independent prediction of aneurysm expansion or clinical outcomes in a model incorporating known clinical risk factors.

**Clinical Trial Registration:**

URL: http://www.isrctn.com. Unique identifier: ISRCTN76413758.

## Abstract 2

Supplemental Digital Content is available in the text.

**Clinical Perspective**

**What Is New?**

- In this proof-of-concept phase 2 study, we demonstrate for the first time that functional imaging of abdominal aortic aneurysms can predict disease progression and clinical events.
- Aortic wall inflammation detected by ultrasmall superparamagnetic particles of iron oxide–enhanced magnetic resonance imaging predicts the rate of aneurysm growth and the risk of aneurysm rupture or repair as well as being associated with all-cause and aneurysm-related mortality.

**What Are the Clinical Implications?**

- Multivariate analysis demonstrated that ultrasmall superparamagnetic particles of iron oxide–enhanced magnetic resonance imaging does not appear to improve risk stratification beyond current predictors of clinical outcome, including ultrasound measures of aneurysm diameter.
- This technique may be a useful adjunctive imaging approach in those with high-risk or borderline aneurysm sizes or those with larger aneurysms, where the balance of risk and benefit is uncertain.
- This approach may also have utility in assessing candidate anti-inflammatory therapies targeted at reducing disease progression.

Abdominal aortic aneurysms have a prevalence of 5% in 65- to 74-year-old men and, when ruptured, are associated with a mortality of ≤90%.^1^ At a population level, ruptured aortic aneurysms are a major cause of death, being the 13th most common cause of death and accounting for >150 000 deaths in 2013.^2^ Preemptive elective open surgical or endovascular repair can be life-saving and is considered when the abdominal aortic aneurysm diameter is >55 mm, is rapidly expanding (≥10 mm/year), or causes symptoms.^3–5^

Abdominal aortic aneurysms are usually associated with no symptoms and are often identified incidentally or as part of an ultrasound-based screening program. Population screening has been established in some countries and is associated with a halving of the mortality associated with abdominal aortic aneurysms.^6,7^ However, continued surveillance of aneurysms is challenging because of the nonlinearity and unpredictability of expansion rates,^8^ although the best current predictor of aneurysm expansion and rupture is the baseline aneurysm diameter.^1,9^ Furthermore, the pathophysiological mechanisms underlying aneurysm expansion remain uncertain, and the role of cellular inflammation and macrophage infiltration has been debated. Last, ≤20% of ruptured abdominal aortic aneurysms are <55 mm in diameter, and 40% of patients with aneurysm diameters between 70 and 100 mm do not experience aneurysm rupture.^10^ Therefore, an unmet clinical need exists to identify more reliable methods of identifying those patients at risk of abdominal aortic aneurysm expansion and rupture,^11,12^ and techniques that assess both the structure and biology of aneurysms hold considerable promise.

Ultrasmall superparamagnetic particles of iron oxide (USPIO) constitute a class of magnetic resonance imaging (MRI) contrast agent taken up by tissue-resident macrophages and can be used to identify cellular inflammation within tissues,^13,14^ including abdominal aortic aneurysms.^15,16^ In a small pilot study of 29 patients with abdominal aortic aneurysm,^15^ we have previously demonstrated that USPIO enhancement on MRI is associated with macrophage infiltration of the abdominal aortic aneurysm wall and more rapid rates of abdominal aortic aneurysm expansion. We therefore aimed to validate these preliminary findings in a larger multicenter cohort of patients and determine whether USPIO-enhanced MRI could predict the rate of abdominal aortic aneurysm expansion and subsequent rates of rupture or surgical repair.

## Methods

### Study Design

The MA^3^RS study (MRI Using Ultrasound Superparamagnetic Particles of Iron Oxide to Predict Clinical Outcome in Patients Under Surveillance for Abdominal Aortic Aneurysms) was a prospective multicenter observational open-label cohort study of patients under routine ultrasound surveillance for abdominal aortic aneurysm. The research design and protocol has been described previously (ISRCTN.com. Unique identifier: ISRCTN76413758).^17^ The study was approved by the local research ethics committee (12/ES/0068), and the use of ferumoxytol was given clinical trial authorization by the Medicines and Healthcare products Regulatory Authority of the United Kingdom (EudraCT Number 2012-002488-25).

### Study Population

Consecutive patients were recruited from 3 centers in Scotland (Royal Infirmary of Edinburgh, Western Infirmary of Glasgow, and Forth Valley Royal Hospital in Larbert) between November 8, 2012, and December 5, 2014. Inclusion criteria were age >40 years, maximum anteroposterior abdominal aortic aneurysm diameter ≥40 mm by abdominal ultrasound, and under ultrasound surveillance as part of routine clinical care. Exclusion criteria included patients with planned repair of abdominal aortic aneurysm, known inflammatory aneurysm, aneurysm arising from a connective tissue disorder, women of childbearing potential, renal failure (estimated glomerular filtration rate ≤30 mL/min/1.73 m^2^), and contraindication to MRI or ferumoxytol. All participants gave written informed consent to participate in the study.

### Study Protocol

**Figure 1.**
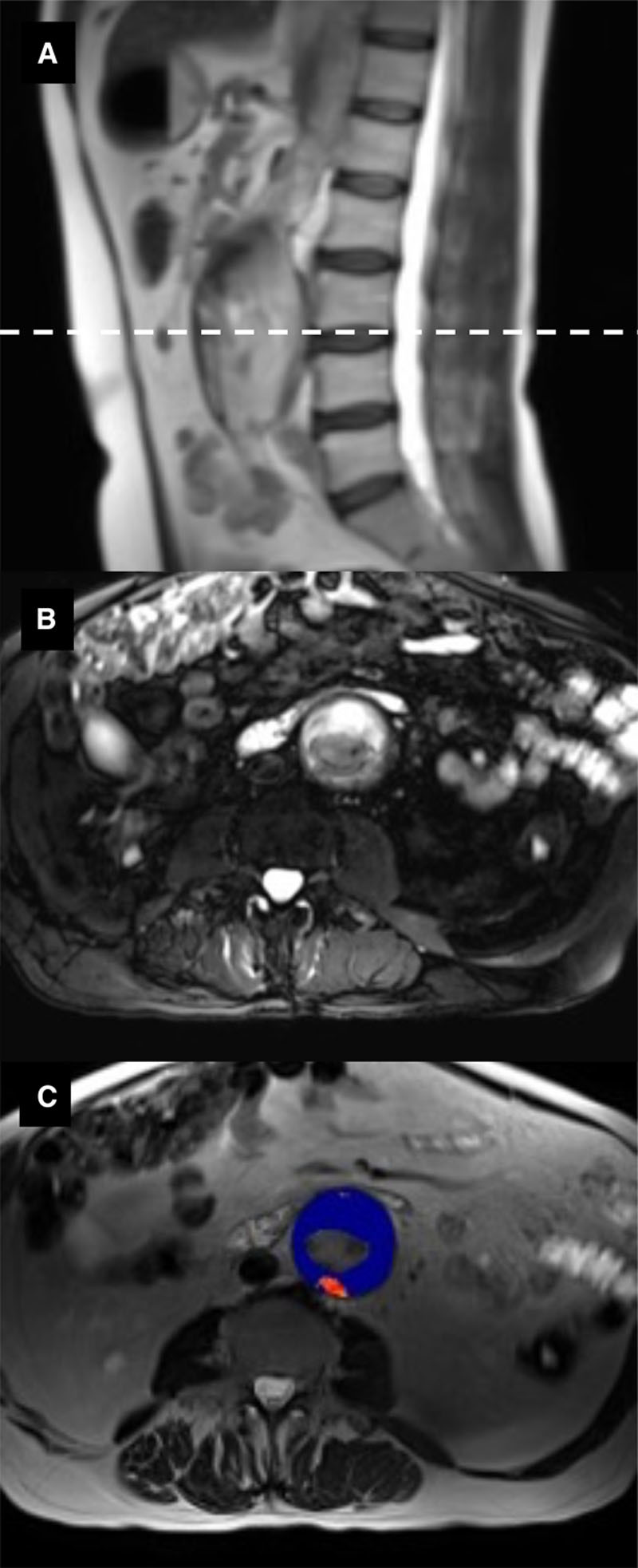
**MRI of abdominal aortic aneurysm.** **A**, T2-weighted HASTE (Half Fourier Acquisition Single Shot Turbo Spin Echo) sequence in the sagittal plane. **B**, Cross-sectional image (dashed line in **A**) using a T2-weighted fat-saturated sequence to highlight intraluminal thrombus (white) within the aneurysm. **C**, T2* map (blue) overlying the T2-weighted HASTE sequence (**B**), demonstrating enhancement of the posterior aneurysm wall with ultrasmall superparamagnetic particles of iron oxide (USPIO) (red).

Participants received a baseline assessment within 6 weeks of the screening abdominal ultrasound. Participant characterization comprised a full clinical assessment, USPIO-enhanced MRI, and computed tomography aortography. The scanning protocols and image analysis techniques have been described previously.^15,17^ In brief, patients underwent a baseline 3T MRI (Magnetom Verio 3T, Siemens Healthcare) before receiving an intravenous infusion of a weight-adjusted dose of USPIO (4 mg/kg of ferumoxytol; Rienso). A second MRI scan was performed 24 to 36 hours after USPIO administration. Two trained observers performed image analysis using bespoke software that compared before and after contrast images using semiautomatic registration. To calculate the degree of USPIO enhancement, color maps were generated to depict the percentage change in T2*, which is the decay constant for the exponential decay of signal over time. Using the predefined threshold of ≥71% change in T2*, each color map was independently classified by 2 trained observers into patients with or without USPIO enhancement (≥10 contiguous voxels^15^) within the wall of the abdominal aortic aneurysm (Figure 1). Discordant classifications were resolved by consensus.

### Clinical Follow-Up

Patients were reviewed every 6 months in the research clinic for ≥24 months. Structured follow-up data were collected on abdominal aortic aneurysm events, hospital admissions, and other relevant clinical data. Clinical events were verified independently using electronic health records and public registry data as described previously.^18,19^ Serial maximum anteroposterior diameters were obtained by ultrasound in dedicated abdominal aortic aneurysm surveillance clinics performed by trained specialist vascular practitioners who were blinded to USPIO-enhanced MRI findings. We have previously reported interobserver coefficient of variation of aortic diameter measurements of 3.5%.^20^ Participants who were unable to attend for subsequent research visits were followed up through electronic health records as described previously.^18,19^

### Clinical End Points and Adjudication

Clinical data from clinic visits, research database, electronic health records, primary care contacts, and the General Register Office were reviewed, and clinical end points were adjudicated by an independent Clinical End Point Committee. The committee members were blinded to the MRI findings. Follow-up was censored on November 21, 2016, or at the time of event.

### Statistical Analysis

The primary end point was the composite of abdominal aortic aneurysm rupture or repair. We estimated that 130 events would be required to have adequate sensitivity to determine the added value of USPIO-enhanced MRI to predict the occurrence of the primary end point. Previous data from the United Kingdom have suggested a 2-year event rate of 41% in patients under surveillance for abdominal aortic aneurysm.^21^ Therefore, we aimed to recruit ≈350 patients, with an expected dropout rate of 10%, resulting in ≥317 patients with 130 events to be included in the final analysis.

Categorical data are presented using counts and percentages, continuous variables are presented using mean±standard deviation, and absolute differences are presented with 95% confidence intervals (CIs). Comparisons in baseline characteristics were made using either a binomial test for proportions in the case of categorical data or by 2-sample *t* test for continuous data. Aneurysm growth rate was determined from serial ultrasound measurements using a linear regression model that was fitted to all available data, and the slope was used to determine the aneurysm growth rate per year. The primary and clinical event end points were assessed by log-rank test and are presented as Kaplan-Meier curves. Cox proportional hazards models were generated to include the baseline covariates of sex, smoking, systolic blood pressure, and baseline aneurysm diameter determined by ultrasound. The additional value of USPIO enhancement was assessed by the c-statistic and net reclassification index.^22–24^ Statistical significance was taken as 2-sided *P*<0.05.

## Results

**Figure 2.**
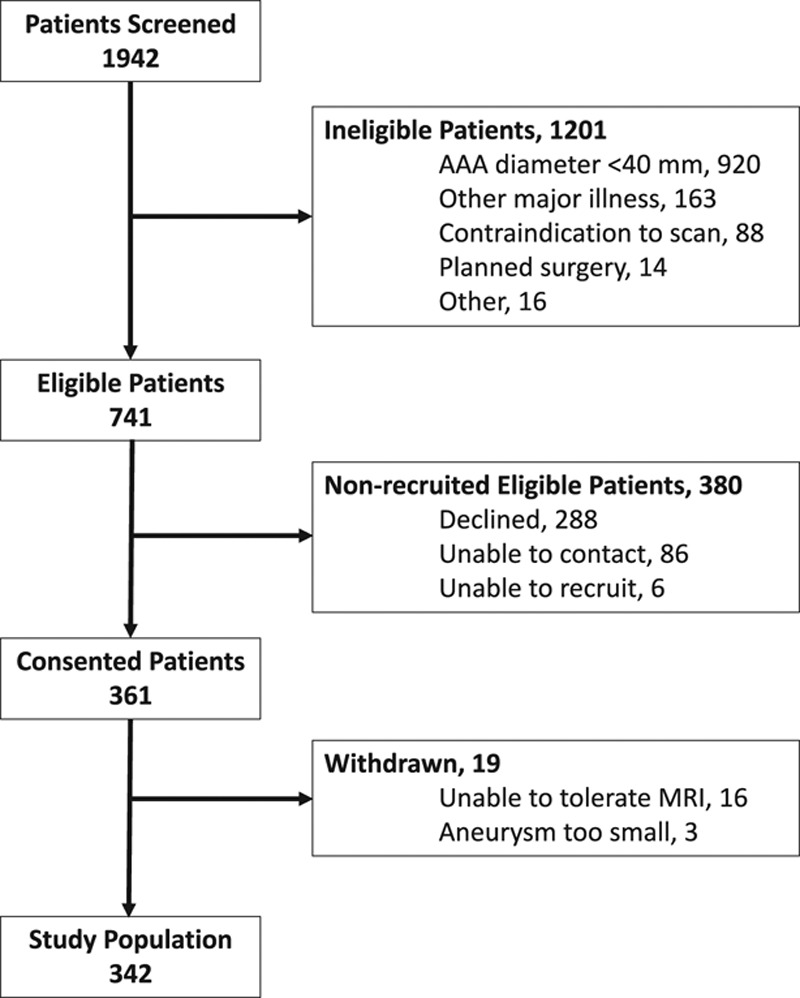
**Consolidated Standards of Reporting Trials (CONSORT) diagram of participant recruitment.**

We screened ≈2000 patients attending the outpatient vascular clinics of the study centers and identified 741 potentially eligible patients, of whom ultimately 361 (48.7%) were recruited into the study (Figure 2). Nineteen patients were subsequently withdrawn predominantly because they were unable to undergo repeated MRI because of claustrophobia. The final study population comprised 342 participants who were predominantly elderly male current or former smokers with hypercholesterolemia and hypertension (Table 1). No serious adverse events or reactions to intravenous ferumoxytol administration occurred; the medication was generally well tolerated by all participants. Mild asymptomatic hypotension possibly related to ferumoxytol was noted in 1 subject but required no action or intervention.

**Table 1.**
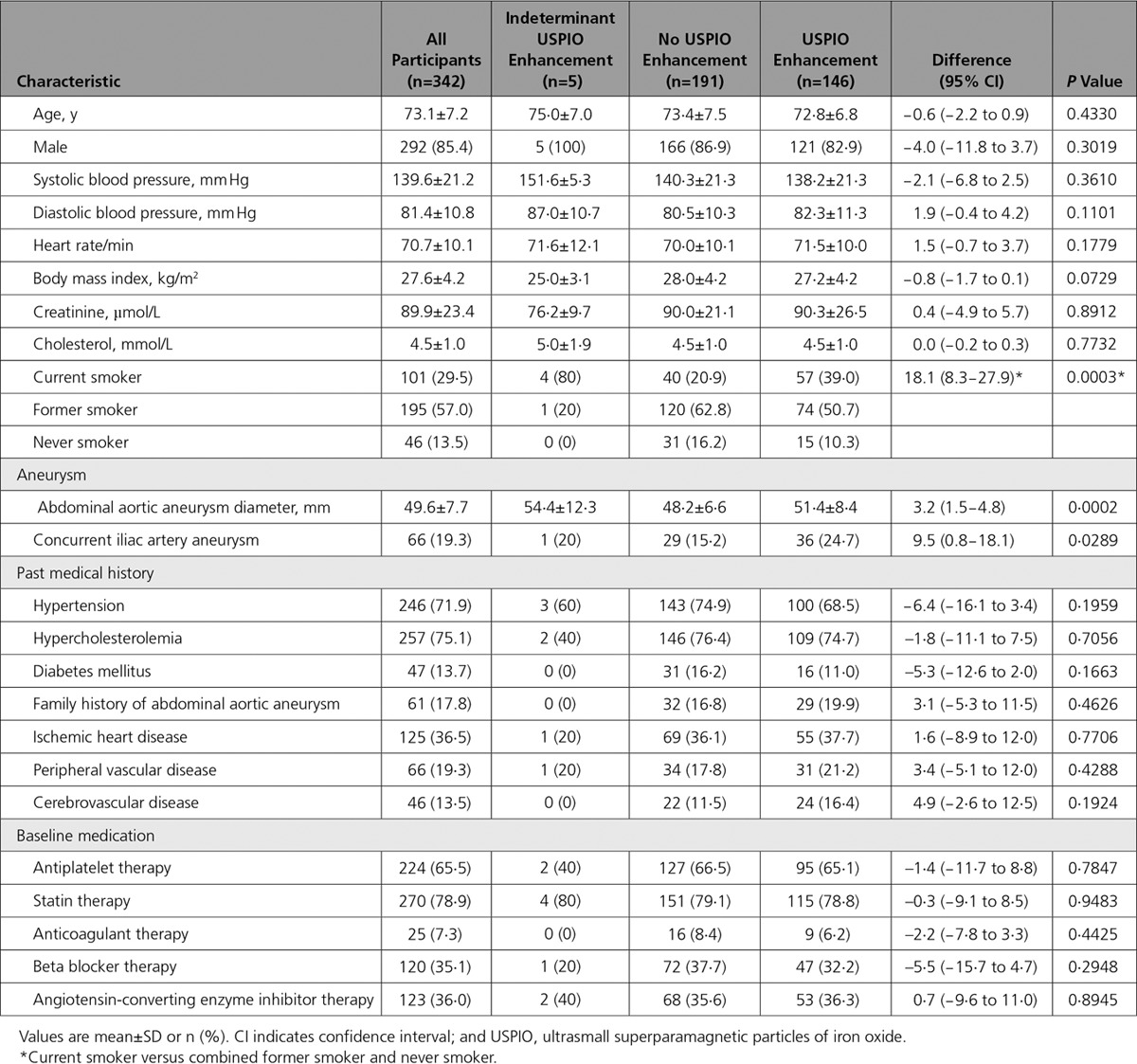
Baseline Characteristics of Participants

USPIO enhancement of the abdominal aortic aneurysm wall was identified in 146 (42.7%) participants, was absent in 191 (55.8%), and was indeterminate in 5 (1.5%). USPIO enhancement was strongly associated with current smoking status as well as baseline abdominal aortic aneurysm diameter and the presence of a common iliac aneurysm (Table 1).

### Aneurysm Growth Rate

On ultrasound, baseline maximum abdominal aortic aneurysm diameter was 49.6±7.7 mm and was slightly larger in patients with USPIO enhancement (Table 1). The abdominal aortic aneurysm growth rate during the trial was 2.8±2.4 mm/year (n=279) and was greater in patients with USPIO enhancement (3.1±2.5 versus 2.5±2.4 mm/year; difference 0.6 mm/year; 95% CI, 0.02−1.2; *P*=0.0424). Current smoking status (*P*=0.0305), but not aneurysm diameter (*P*=0.1853), baseline systolic blood pressure (*P*=0.6994), or USPIO enhancement (*P*=0.1993), was an independent predictor of aneurysm growth rate.

### Clinical Follow-Up

All participants were followed up for a mean of 1005±280 days. Overall, the primary end point occurred in 140 (40.9%) subjects with 17 abdominal aortic aneurysm ruptures and 126 abdominal aortic aneurysm repairs (Table 2): 3 subjects underwent repair after rupture. In all, 48 (14.0%) deaths occurred, of which one third were related to abdominal aortic aneurysm (17 [35.4%]), and a quarter were related to other cardiovascular causes (12 [25.0%]).

**Table 2.**
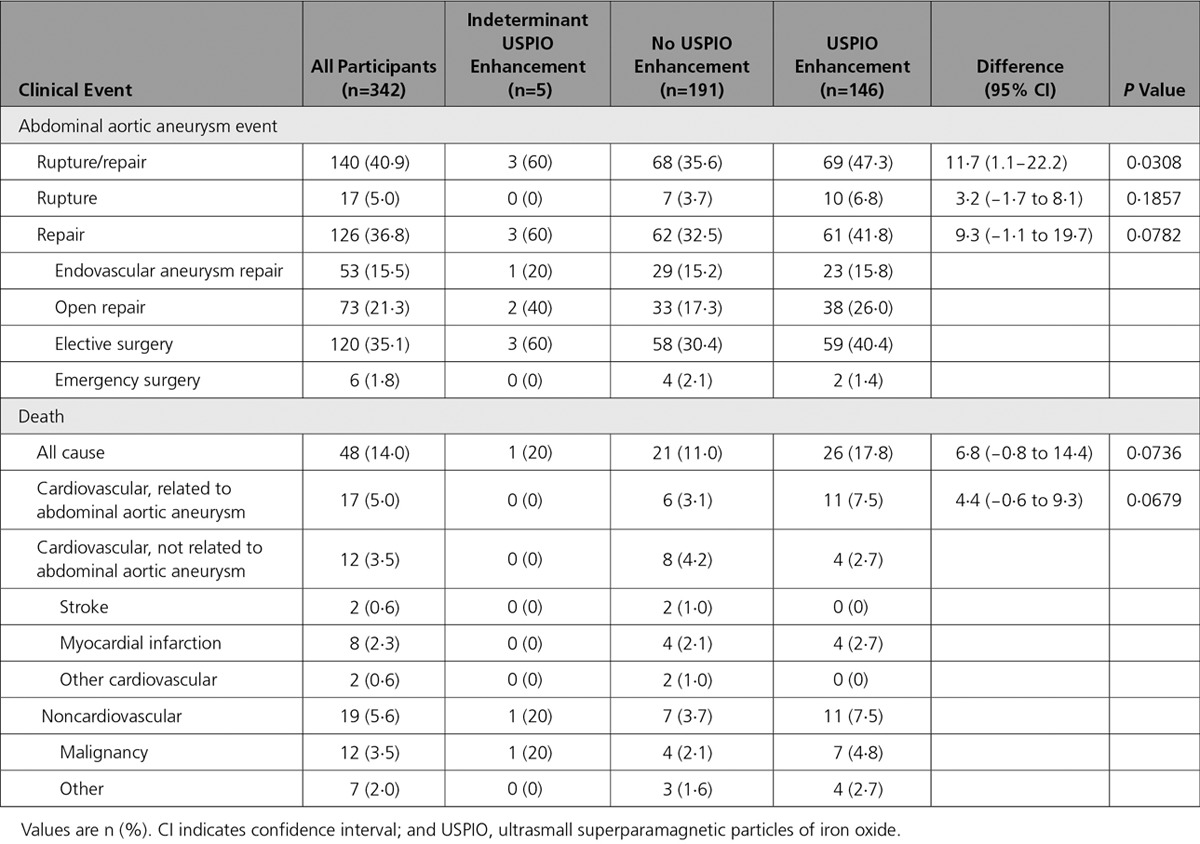
Clinical Outcome Events in All Patients

### Rupture or Repair

**Figure 3.**
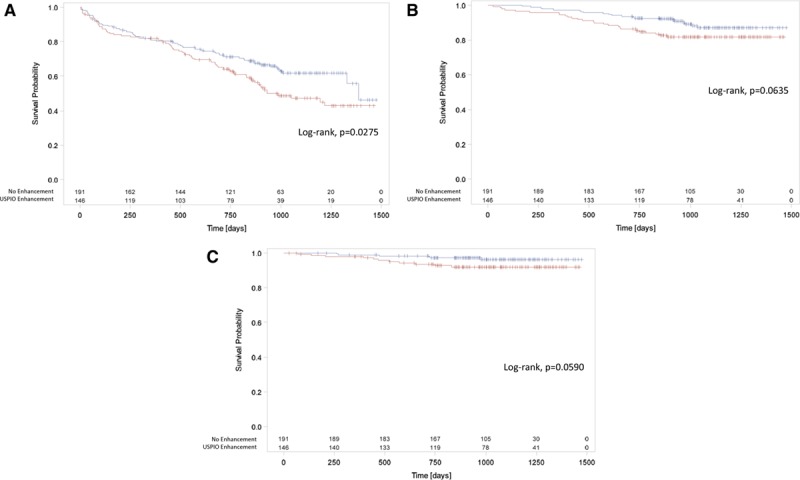
**Major clinical events in the MA3RS study.** Abdominal aortic aneurysm rupture or repair (primary end point) (**A**), all-cause mortality (**B**), and aneurysm-related mortality (**C**) in participants with (red) and without (blue) ultrasmall superparamagnetic particles of iron oxide (USPIO) enhancement of the aneurysm wall. Cross-hairs represent individual censoring.

The primary end point occurred more frequently in participants with USPIO enhancement of abdominal aortic aneurysm (69/146=47.3% versus 68/191=35.6%; difference 11.7%, 95% CI 1.1–22.2%; *P*=0.0308) and was associated with a reduced event-free survival (*P*=0.0275; Figure 3). This finding was consistent for both components of the end point (Table 2). In contrast with female sex (hazard ratio [HR], 0.952; 95% CI, 0.589−1.540; *P*=0.8413) and systolic blood pressure (HR, 0.997; 95% CI, 0.988−1.005; *P*=0.4492), baseline abdominal aortic aneurysm diameter (HR, 1.077; 95% CI, 1.061−1.094; *P*<0.0001), and current smoking habit (HR, 1.464; 95% CI, 1.001–2.120; *P*=0.0433) were the main predictors of the primary end point. The addition of USPIO enhancement to the model (HR, 1.003; 95% CI, 0.700–1.439; *P*=0.9849) did not improve the prediction of events (c-statistic, 0.7924−0.7926) or the unconditional net reclassification (−13.5%; 95% CI, −36.4 to 9.3). This finding was true for both components of the end point: (1) aneurysm rupture, c-statistic, 0.6317 to 0.6304, and net reclassification, 29.9% (95% CI, −22.0 to 81.9); and (2) aneurysm repair, c-statistic, 0.8000 to 0.7996, and net reclassification, −9.9% (95% CI, −33.4 to 13.7).

All-cause and abdominal aortic aneurysm-related death appeared to be more frequent in participants with USPIO enhancement of the abdominal aortic aneurysm (Table 2 and Figure 3) but fell short of statistical significance.

In post hoc analysis, we explored whether USPIO enhancement varied according to aneurysm size. We dichotomized the population at the mean diameter into smaller (diameter 40–49 mm; n=187) and larger (diameter ≥50 mm; n=155) aneurysms. The rate of USPIO enhancement was lower in patients with smaller aneurysms: 65 (35.1%) versus 81 (53.3%) in those with larger aneurysms, difference 18.2% (95% CI, 7.7–28.9; *P*=0.0008). However, in patients with smaller aneurysms, USPIO enhancement was associated with a doubling in the rate of repair or rupture without an effect on mortality (Table 3), whereas in those with larger aneurysms, the reverse occurred, with a more than doubling of mortality but no effect on the primary end point (Table 4).

**Table 3.**
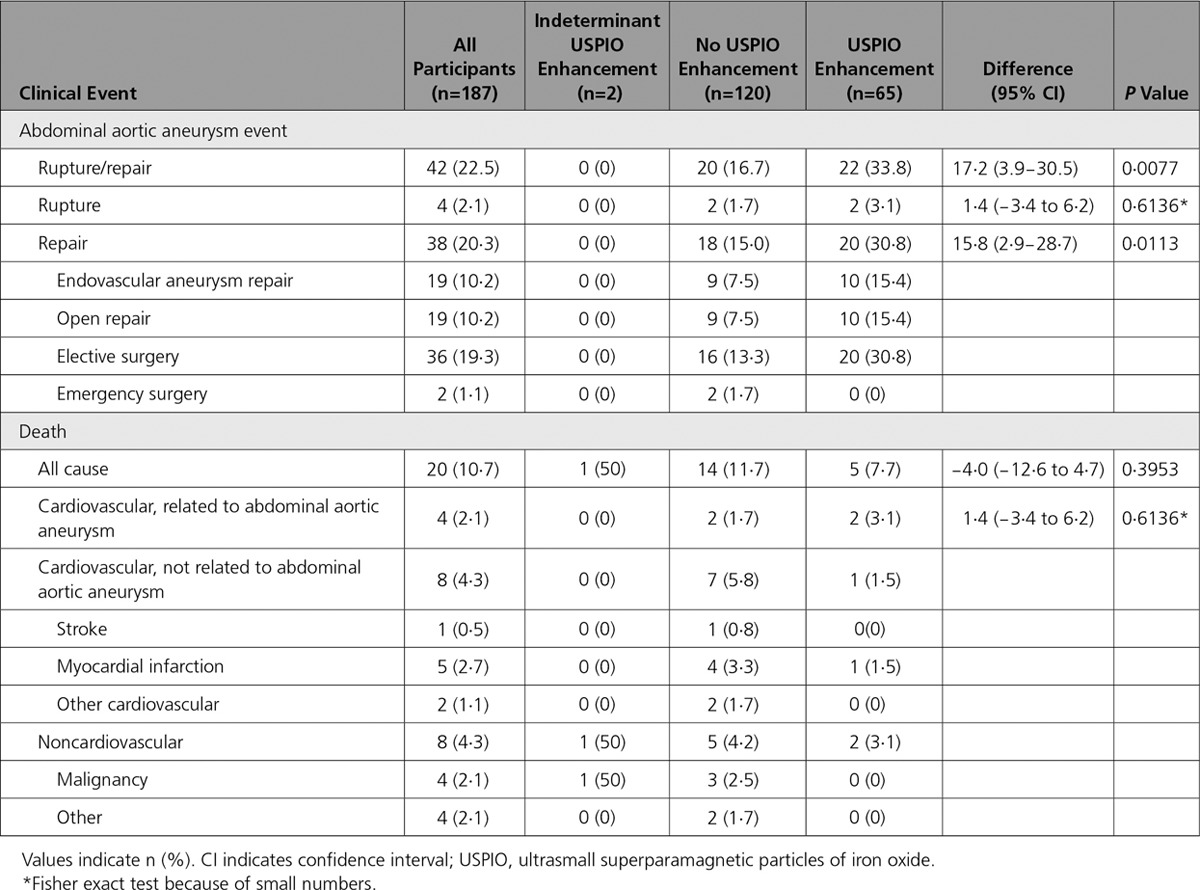
Clinical Outcome Events in Patients With Small Aneurysms (Diameter, 40–49 mm)

**Table 4.**
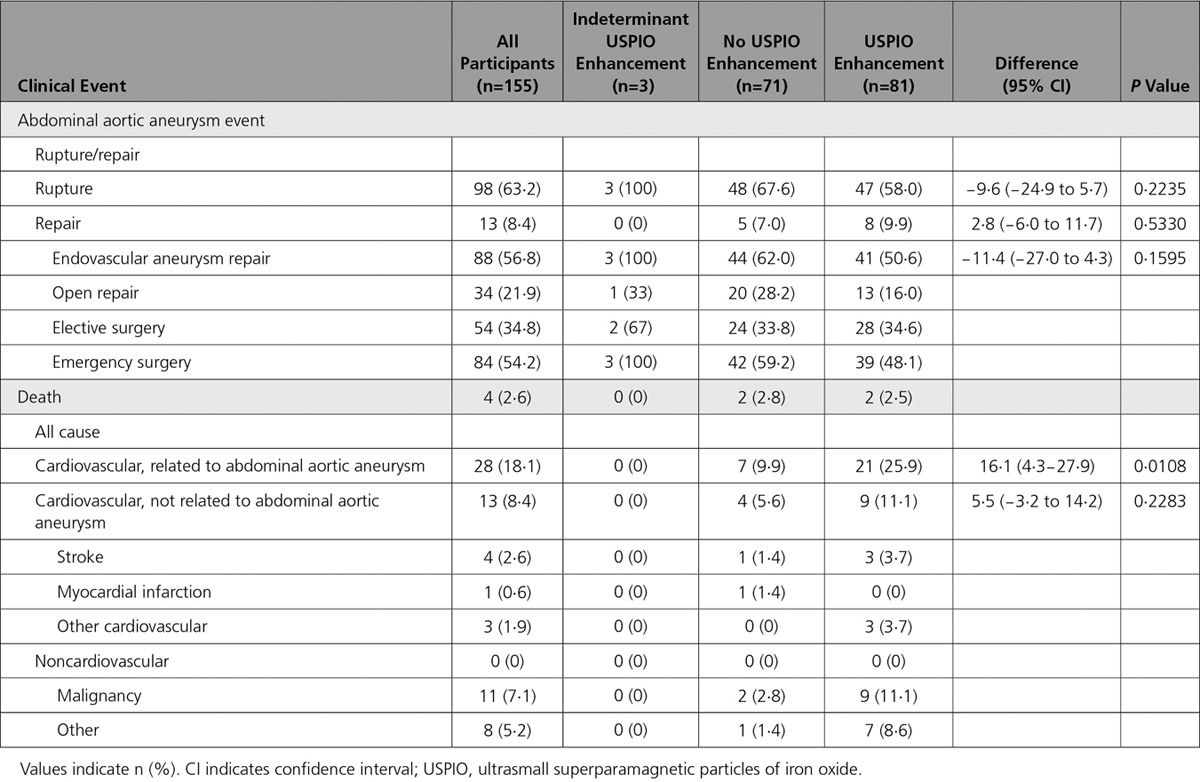
Clinical Outcome Events in Patients With Large Aneurysms (Diameter ≥50 mm)

## Discussion

In a prospective multicenter observational cohort study, we have demonstrated that USPIO-enhanced MRI predicts the rate of aneurysm expansion as well as the future risk of abdominal aortic aneurysm rupture or repair. This prospective clinical study of MRI in patients with abdominal aortic aneurysms is the largest and the first to report on an imaging technique that identifies cellular inflammation and predicts disease progression and outcome. This study suggests a central role of cellular inflammation in the pathophysiology, progression, and outcome of abdominal aortic aneurysm disease.

Abdominal aortic aneurysm expansion is driven by several potential pathogenetic mechanisms that are associated with inflammation and tissue degradation.^1,11^ Macrophages are central to many of these processes,^25^ and their depletion appears to prevent aneurysm formation or progression in preclinical models of abdominal aortic aneurysm.^26^ Noninvasive in vivo imaging of tissue-resident macrophages would therefore seem an intuitive and promising approach in patients with abdominal aortic aneurysm, but until now this approach has not been prospectively tested in large clinical cohorts.^11,12,27^ We here report the first study in a large clinical cohort to image tissue-resident macrophages with USPIO-enhanced MRI, and we demonstrate that USPIO enhancement is associated with more rapid abdominal aortic aneurysm growth rates and adverse clinical outcomes. This finding provides strong support for the concept that imaging the biology of abdominal aortic aneurysm may be a promising new approach to risk stratify and manage patients with this disease.^11^

The rate of abdominal aortic aneurysm growth has previously been shown to be predicted by smoking status, aneurysm size, and the presence of common iliac aneurysms.^9,28^ Indeed, a smoking habit is the principal modifiable risk factor for abdominal aortic aneurysm progression and rupture and is the main focus of lifestyle modification in these patients. We here demonstrate that USPIO-enhanced MRI is associated with these 3 risk factors. In particular, current smoking was an independent risk factor for abdominal aortic aneurysm growth, and intriguingly, USPIO enhancement was twice as frequent in current smokers. We know that smoking promotes inflammation, macrophage-mediated injury, and vascular dysfunction.^29–31^ This finding suggests a potential mechanistic link between smoking and macrophage-driven abdominal aortic aneurysm inflammation. Indeed, components of cigarette smoke, such as 3,4-benzopyrene, promote macrophage infiltration of abdominal aortic aneurysm, leading to increased matrix metalloproteinase expression and vascular smooth muscle apoptosis.^32^ Using adoptive transfer experiments, Jin and colleagues^33^ have further shown that in vivo exposure of leukocytes to smoke can accelerate the progression of aneurysm disease in smoke-free animals. In this context, our USPIO data suggest that macrophage-mediated inflammation may be the mechanistic link to explain the association between smoking and disease progression in patients with abdominal aortic aneurysm.

The primary end point of the study was the rate of abdominal aortic aneurysm rupture or repair. Although the rate was higher in patients with USPIO enhancement on MRI, it was not independent of known predictors of outcome, including baseline abdominal aortic aneurysm diameter and smoking habit. Indeed, incorporation of USPIO-enhanced MRI did not improve the discrimination of a model incorporating these known clinical risk factors. This finding likely reflects the mutual interdependence and potential causal association of these factors, namely, that USPIO enhancement highlights areas of smoking-induced cellular inflammation within the aneurysm, which causes more rapid expansion and increase in the aneurysm diameter, leading to aneurysm rupture or triggering of the threshold for repair.

Ultrasound measurements of abdominal aortic aneurysm diameter are the mainstay of clinical management and the principal determinant of the timing of elective surgical repair. Therefore, their dominant effect on the primary end point is perhaps not surprising, especially as most events were because of elective surgical repair. Given that the clinicians were blind to the results of the USPIO-enhanced MRI, it would be challenging to demonstrate that it could lead to any changes in the rate of elective surgical repair. We therefore explored other measures of outcome that were independent of elective surgical repair. We found that USPIO enhancement appeared to be greater in those with emergent abdominal aortic aneurysm-related events, including abdominal aortic aneurysm rupture and abdominal aortic aneurysm-related mortality, although the absolute number of events was small and fell just short of achieving statistical significance. Given the small number of emergent events, our study did not have sufficient power to determine whether USPIO enhancement could provide clinically useful information that could independently predict emergent events. However, post hoc analyses did suggest that USPIO-enhanced MRI predicted overall mortality in patients with larger aneurysms.

Although USPIO-enhanced MRI was not an independent predictor of outcome across the whole study population, it identified aneurysm disease activity, correlated with rates of aneurysm expansion, and appeared to predict clinical outcome, including rupture and death. If future studies confirm the utility of USPIO-enhanced MRI, how would it be applied in the clinic? For some patients, treatment decisions are not straightforward. For example, abdominal pain in a patient with an aortic aneurysm may be because of other abdominal pathology and not the aneurysm. Urgent repair may be unhelpful in such circumstances and associated with considerable risk. Furthermore, decisions to undertake surgical repair can be challenging in those with high-risk or morphologically atypical aneurysms <55 mm, those with borderline aneurysm sizes of 50 to 55 mm (especially in women), or those with larger aneurysms where the balance of risk and benefit is uncertain. Additional information regarding disease activity that is tied to disease progression and adverse clinical outcome may be helpful in guiding such decisions. The value of USPIO-enhanced MRI may also differ according to aneurysm size, with the prediction of future aneurysm repair greater in patients with smaller aneurysms and the future mortality risk more marked in those with larger aneurysms. Although not directly tested here, USPIO-enhanced MRI may assist the clinician in making these difficult management decisions that are associated with significant potential benefits and hazards. This requires further investigation.

No definitive medical treatments can impact disease progression in this serious and potentially fatal condition. Novel anti-inflammatory or other disease-modifying therapies are potential interventions that could address this unmet clinical need. USPIO-enhanced MRI would provide a useful surrogate biomarker of efficacy in such early proof-of-concept clinical trials. Reduction in USPIO enhancement would be predicted to correlate with reduced cellular inflammation within the aneurysm and consequently reduced rates of expansion. This theory merits further investigation.

Our study has a number of strengths. It was a multicenter prospective observational cohort study, which ensured blinding of the USPIO-enhanced MRI findings from the patients, vascular technicians, and attending clinicians, and was therefore independent of clinical decision making. It was an adequately sized phase 2 proof-of-concept trial that was ≈10-fold larger than previous studies in this area.^15,34^ The study also achieved its predicted event rates and met its primary end point, although not independent of known clinical predictors. However, the inclusion of elective surgical repair in the primary end point generates some challenges in interpretation because of the ultrasound- and diameter-guided decision making for elective surgical repair. The prediction of emergent events appears promising but will require a much larger study with greater power to confirm these findings. Finally, USPIO-enhanced MRI is resource intensive and was not possible in a small number of patients because of contraindications or claustrophobia. However, it was a feasible, safe, and deliverable clinical technique that was well tolerated in the vast majority of patients with no serious adverse effects of the MRI or contrast medium. Moreover, we have demonstrated its applicability across multiple sites, and we have developed robust computer algorithms and image analysis techniques that enable automated reporting of USPIO enhancement, lending itself to immediate clinical application.

In conclusion, in a multicenter prospective observational cohort study, we have demonstrated that USPIO-enhanced MRI predicts the rate of aneurysm expansion and the risk of abdominal aortic aneurysm rupture and repair. Although this study does not provide independent prediction of aneurysm expansion or clinical outcomes in a model incorporating known clinical risk factors, it is the first demonstration of a cellular imaging technique that can predict clinical events in patients with abdominal aortic aneurysm. Whether clinical outcomes can be improved by treatment decisions on the basis of this novel imaging approach remains to be established.

## Acknowledgments

The Edinburgh Clinical Research Facility and Edinburgh Imaging Facility are supported by National Health Service Research Scotland. The authors acknowledge the support of Karen Gallagher, Janice Taylor, Hayley Cuthbert, Annette Cooper, and David Brian during the conduct of this study. The MA^3^RS Study Investigators contributed to the conception or design of the work; and acquisition, analysis, or interpretation of data of the work. Ms Rachel O. Forsythe and Dr Newby wrote the first draft of the article, The MA^3^RS Study Investigators were involved in drafting the article and revising it and have given final approval of the version to be published, and the MA^3^RS Study Investigators are accountable for the work.

## Sources of Funding

This study was funded by the Medical Research Council and managed by the National Institute of Healthcare Research on behalf of the Medical Research Council-National Institute of Healthcare Research partnership (National Institute of Healthcare Research Efficacy and Mechanism Evaluation Program: funding reference 11/20/03). Dr Newby is supported by the British Heart Foundation (CH/09/002, RE/13/3/30183, RM/13/2/30158) and is the recipient of a Wellcome Trust Senior Investigator Award (WT103782AIA). Olivia B.M. McBride is supported by the Academic Department of Military Surgery and Trauma.

## Disclosures

A patent (US 9275432 B2) held by the University of Edinburgh has been filed relating to the registration of medical images generated as part of this study. The Medical Research Council played no role in developing the study design; the collection, analysis, and interpretation of data; writing of the report; or the decision to submit the article for publication. The views expressed in this article are those of the authors and not necessarily those of the Medical Research Council, National Health Service, National Institute of Healthcare Research, or Department of Health.

## Supplementary Material

## Appendix

### The MA^3^RS Study Investigators

Chief Investigator: David Newby.

Trial Research Fellows: Rachael Forsythe, Olivia McBride, Jennifer Robson, Alex Vesey.

Study sites: Royal Infirmary of Edinburgh: Roderick Chalmers, Paul Burns, O James Garden, David Newby, Rachael Forsythe, Olivia McBride, Jennifer Robson, Scott Semple, Marc Dweck, Calum Gray, Tom MacGillivray, Chengjia Wang, Yolanda Georgia Koutraki, Neil Mitchard, Annette Cooper, Edwin van Beek, Graham McKillop, Weiyang Ho, Liz Fraser, Hayley Cuthbert, Peter Hoskins, Barry Doyle, Noel Conlisk. Western Infirmary, Glasgow: Wesley Stuart, Colin Berry, Alex Vesey, Giles Roditi, Laura Murdoch. Forth Valley Royal Hospital: Richard Holdsworth, Emma Scott.

Edinburgh Clinical Trials Unit: Lynsey Milne, Fiona Strachan, Fiona Wee, Katherine Oatey, Catriona Graham, Gordon Murray, Garry Milne, Marise Bucukoglu, Kirsteen Goodman.

Clinical Endpoint Committee: Jakub Kaczynski, Anoop Shah, Andrew Tambyraja.

The MA^3^RS Study Steering Committee: Julie Brittenden (chair), Graeme Houston, Robert Lambie, John Norrie, Olivia McBride, Rachael Forsythe, David Newby, Graham McKillop, Scott Semple, Paul Burns, Colin Berry, Gordon Murray, Fiona Wee.
