# Supplementary material for: Aortic Wall Inflammation Predicts Abdominal Aortic Aneurysm Expansion, Rupture, and Need for Surgical Repair
Source: Circulation. 2017 Aug 28;136(9):787–97. doi: 10.1161/CIRCULATIONAHA.117.028433 (PMC5571881; doi:10.1161/CIRCULATIONAHA.117.028433)
Supplement: Supplementary file 1 [file cir-136-787-s001.pdf]

The MA^{3}RS Study Investigators

Chief Investigator: David Newby.

Trial Research Fellows: Rachael Forsythe, Olivia McBride, Jennifer Robson, Alex Vesey.

Study sites: Royal Infirmary of Edinburgh: Roderick Chalmers, Paul Burns, O James Garden, David Newby, Rachael Forsythe, Olivia McBride, Jennifer Robson, Scott Semple, Marc Dweck, Calum Gray, Tom MacGillivray, Chengjia Wang, Yolanda Georgia Koutraki, Neil Mitchard, Annette Cooper, Edwin van Beek, Graham McKillop, Weiyang Ho, Liz Fraser, Hayley Cuthbert, Peter Hoskins, Barry Doyle, Noel Conlisk. Western Infirmary, Glasgow: Wesley Stuart, Colin Berry, Alex Vesey, Giles Roditi, Laura Murdoch. Forth Valley Royal Hospital: Richard Holdsworth, Emma Scott.

Edinburgh Clinical Trials Unit: Lynsey Milne, Fiona Strachan, Fiona Wee, Katherine Oatey, Catriona Graham, Gordon Murray, Garry Milne, Marise Bucukoglu, Kirsteen Goodman.

Clinical Endpoint Committee: Jakub Kaczynski, Anoop Shah, Andrew Tambyraja.

The MA^{3}RS Study Steering Committee: Julie Brittenden (chair), Graeme Houston, Robert Lambie, John Norrie, Olivia McBride, Rachael Forsythe, David Newby, Graham McKillop, Scott Semple, Paul Burns, Colin Berry, Gordon Murray, Fiona Wee.
